# Effect of olive leaf incorporation in animal feed on broiler turkey (*Meleagris gallopavo*) growth performance, welfare, oxidative status, and blood and biochemical serum parameters

**DOI:** 10.5194/aab-67-163-2024

**Published:** 2024-04-17

**Authors:** Ahmed Sadoudi, Asma Ait-Kaki, Yuva Bellik, Leghel Touazi, Krimou Yahi, Mokrane Iguer-Ouada, Jean-Luc Hornick, Nassim Moula

**Affiliations:** 1 Department of Veterinary Management of Animal Resources, Faculty of Veterinary Medicine, University of Liège, 4000 Liège, Belgium; 2 Department of Biology, Faculty of Sciences, M'Hamed Bougara University, 35000 Boumerdès, Algeria; 3 Faculty of Life and Nature Sciences, Mohamed El Bachir El Ibrahimi University, 34000 Bordj Bou Arreridj, Algeria; 4 Department of Agronomy, Faculty of Nature and Life Sciences, Ferhat Abbas University of Setif, El Bez, 19000 Setif, Algeria; 5 Office of hygiene of Azeffoun, Commune d'Azeffoun, 15010 Tizi Ouzou, Algeria; 6 Associated Laboratory in Marine Ecosystems and Aquaculture, Department of Biological Sciences of the Environment, Faculty of Nature and Life Sciences, University of Béjaïa, 06000 Béjaïa, Algeria; 7 Animal Facilities, University of Liège, 4000 Liège, Belgium

## Abstract

This work investigates how incorporating olive leaves (OLs) (*Olea europaea*) into turkeys' (*Meleagris gallopavo*) diets affects their performance, welfare, blood biochemical parameters, and oxidative status of those reared in intensive farming conditions. The potential of this ingredient was assessed by comparing two dietary incorporation rates of olive leaves, 3 % and 6 %, in three commercial feeds corresponding to three growth phases over 15 weeks where feed was provided ad libitum. A total of 270 animals (broiler medium strain) were used. They were divided into three groups: the OL0, OL3, and OL6 regimens with OL incorporation rates of 0 %, 3 %, and 6 %, respectively. Animals were allocated to three pens of approximately 15 m ${}^{2}$ of surface area; each pen had 30 animals, with a density of three turkeys per square meter. Throughout the rearing period, the diet had no effect on bird mortality. Olive leaves boosted growth rates. Indeed, after the experiment, the OL3 group had significantly higher weights than the OL6 and OL0 groups, which had the lowest feed conversion ratio. Furthermore, after bleeding, the weights and evisceration was significantly ( p < 0.05) higher in the OL3 and OL0 groups compared to the OL6 group. However, OL0 turkeys had significantly higher spleen, heart, and abdominal fat weights than OL3 and OL6 turkeys. The three experimental groups had no significant differences ( p>0.05 ) in carcass yield or gizzard or liver weights. OL supplementation improved oxidative status but had no effect on animal welfare or blood biochemical parameters, with the exception of the mean corpuscular hemoglobin concentration (MCHC), which was significantly ( p < 0.05) lower in the OL3 group than in the OL0 and OL6 groups. Except for the mean cholesterol level, which was significantly ( p < 0.05) lower in the OL3 and OL6 (1.29 g L ${}^{-1}$ ) groups compared to the OL0 group, and the albumin level, which was higher in the OL0 group compared to the OL6 group, no significant effect was observed on biochemical serum parameters. Thus, a 3 % OL supplementation in the turkey diet appears promising for improving the bird's growth performance.

## 1 Introduction

*Olea europaea* is a common plant in the Mediterranean region, including Algeria, and is highly valued in the food, therapeutic, pharmaceutical, and cosmetics industries (Markhali et al., 2020). Olive leaves (OLs) are an important byproduct of olive cultivation, accounting for 10 % of total olive harvest weight and 25 kg per tree during pruning (Khemakhem et al., 2017). Olive leaves can be added in small amounts to poultry feed mixtures, because animals can consume large amounts of antinutritional substances such as lignins, tannins, Cu, and polyphenols. Furthermore, olive leaves contain high levels of structural carbohydrates, which are difficult for non-ruminants to digest (Al-Harthi, 2017). The beneficial effects of olives on health, welfare, and performance, on the other hand, are primarily attributed to polyphenols such as oleuropein, hydroxytyrosol, and tyrosol, which have been shown in animal studies to have antimicrobial, antiatherogenic, antioxidant, anti-inflammatory, anticoagulant, antihypertensive, hypolipidemic, and anticancer properties (Manca et al., 2020). Algerian turkey (*Meleagris gallopavo*) farming has remained traditional since the country's independence in 1962 until the 1990s. In 2009, the FAO estimated that there were 70 000 local turkeys.

Algerian local turkeys were primarily raised in extensive systems, and their growth and reproductive performance data were unknown (Feliachi, 2003; Ferrah et al., 2003). These local populations remained very small in the backyards of some eastern regions, including Oum El Bouaghi, Batna, and Constantine. According to Ferrah et al. (2003), turkey population traits in southern and northern Algeria are similar. Algerian local turkeys have high legs, a slender shape, a long neck, a low crest, and voluminous barbels. These populations are divided into black, tan, and red phenotypes (Halbouche et al., 2010). In Algeria, poultry and turkey farming rely on imported inputs, creating a near-total reliance on the domestic market.

Furthermore, fluctuating international raw material prices (corn, soybeans) raise production costs and cause fluctuations in finished-product selling prices. To address these issues and, as a result, reduce turkey production costs, the current study aims to investigate the effect of incorporating olive leaves (locally abundant and considered wastes in olive oil production) in the *Meleagris gallopavo* feed. The feasibility and interest of using olive leaves in this study were assessed by testing their effects on animal zootechnical growth performance (live weight, conversion index, slaughter yield), turkey welfare and oxidative stress, and hematological and biochemical status.

## 2 Materials and methods

### 2.1 Study sites and periods

The study was conducted at a privately-owned farm in Aïn Turk, Bouïra province, Algeria, for 15 weeks.

### 2.2 Plant extracts preparation

The olive leaves were harvested during the olive harvest season (November 2019–January 2020) in the Azazga district (Tizi Ouzou province), 130 km east of Algiers. The harvested leaves were dried in the shade for several days before ground into powder and stored in paper bags. The analytical composition of the olive leaves used in this study was reported by Ait-Kaki et al. (2018) (Table 1).

**Table 1:** Nutritional composition of olive leaves.

Analytical value of olive leaves (g kg ${}^{-1}$ )	
Dry matter	816
Crude ash	77
Total nitrogen	118
Crude cellulose	187
Soluble minerals (g kg ${}^{-1}$ )	
Calcium	18.1
Phosphorus	1.4
Potassium	7.6
Sodium	0.2
Magnesium	2.9

### 2.3 Animal feed

The feed was distributed into three phases: start-up (0–3 weeks), growth period 1 (4–6 weeks), and growth period 2 (7–15 weeks). Turkeys were divided into three groups: OL0 (control group fed commercial feed), OL3 (commercial feed supplemented with 3 % olive leaves), and OL6 (commercial feed supplemented with 6 % olive leaves). During the three rearing stages, the animals were fed ad libitum and had free access to water. Every 2 weeks, the leftover feed per pen was weighed and recorded to determine the true consumption of the turkeys. Tables 2–4 show the feed composition for the three rearing phases.

**Table 2:** Percentages of feed-start ingredients distributed to the control and experimental groups (on a dry matter basis).

Ingredients	(%)
Soybean oil cake	49.6
Corn	39.7
Soybean oil	1.94
Calcium phosphate	3.15
Calcium carbonate	0.99
Di Methionin	0.27
Lysine	0.25
Premix start ${}^{*}$	0.09
Salt	0.45
Bran meal	0.27
Olive leaves analytical composition (g kg ${}^{-1}$ )	
Metabolizable energy (kcal kg ${}^{-1}$ )	2617
Crude protein (%)	26.3
Ca (%)	1.38
P (%)	1.00
Lysine (%)	1.71
Methionine (%)	0.64
Premix start 1 composition per kg	
Vitamin A	1 300 000 IU
Vitamin D ${}_{3}$	400 000 IU
Vitamin E	10 000 mg
Vitamin B ${}_{1}$	400 mg
Vitamin K ${}_{3}$	400 mg
Vitamin B ${}_{2}$	1000 mg
Vitamin B ${}_{6}$	700 mg
Vitamin B ${}_{12}$	4 mg
Niacin (ppm)	7500 mg
Pantothenic acid	2500 mg
Biotin	30 mg
Folic acid	7000 mg
Iron	875 mg
Copper	7000 mg
Zinc	12 250 mg
Manganese	175 mg
Iodine	70 mg
Cobalt	35 mg
Selenium	250 000 mg
Methionine	250 000 mg
Lysine	60 000 mg
Choline chloride	60 000 mg
Threonine	100 000 IU
Antioxidant	10 000 IU

${}^{*}$ The composition of premix start per kg is shown in the lower section of this table. IU is international unit.

**Table 3:** Percentages of feed-growth 1 ingredients given to the control and experimental groups (on a dry matter basis).

Ingredients (%)	Control group	OL3 %	OL6 %
Soybean oil cake	47.7	47.3	47.0
Corn	42.1	40.1	38.6
Soybean oil	2.70	2.98	3.16
Calcium phosphate	2.80	2.89	2.79
Calcium carbonate	0.87	0.78	0.63
Di Methionin	0.21	0.21	0.22
Lysine	0.25	0.27	0.29
Premix growth 1 ${}^{*}$	0.45	0.45	0.45
Salt	0.27	0.27	0.27
Bran meal	2.65	1.75	0.6
Olive leaves	0	3	6
Olive leaves analytical composition (g kg ${}^{-1}$ )			
Metabolizable energy (kcal kg ${}^{-1}$ )	2713	2713	2713
Crude protein (%)	25.4	25.5	25.5
Ca (%)	1.26	1.26	1.26
P (%)	0.94	0.93	0.90
Lysine (%)	1.63	1.63	1.63
Methionine (%)	0.59	0.59	0.59
Premix growth 1 composition per kg			
Vitamin A	800 000 IU	–	–
Vitamin D ${}_{3}$	370 000 IU	–	–
Vitamin E	5000 mg	–	–
Vitamin B1	200 mg	–	–
Vitamin K3	200 mg	–	–
Vitamin B2	500 mg	–	–
Vitamin B6	400 mg	–	–
Vitamin B12	2 mg	–	–
Niacin (ppm)	6500 mg	–	–
Pantothenic acid	1600 mg	–	–
Biotin	25 mg	–	–
Folic acid	200 mg	–	–
Iron	7000 mg	–	–
Copper	875 mg	–	–
Zinc	7000 mg	–	–
Manganese	12 250 mg	–	–
Iodine	175 mg	–	–
Cobalt	70 mg	–	–
Selenium	35 mg	–	–
Methionine	200 000 mg	–	–
Lysine	200 000 mg	–	–
Choline chloride	50 000 mg	–	–
Threonine	100 000 mg	–	–
Antioxidant	10 000 mg		

${}^{*}$ The composition of premix growth 1 per kg is shown in the lower section of this table.

**Table 4:** Percentages of feed-growth 2 ingredients given to the control and experimental groups (on a dry matter basis).

Ingredients (%)	Control group	OL3 %	OL6 %
Soybean oil cake	40.3	41.1	39.7
Corn	48.8	48.1	46.4
Soybean oil	3.53	3.43	3.67
Calcium phosphate	2.57	2.56	2.52
Calcium carbonate	0.78	0.60	0.56
Di Methionin	0.24	0.26	0.28
Lysine	0.16	0.17	0.22
Premix growth 2 ${}^{*}$	0.45	0.45	0.45
Salt	0.27	0.27	0.27
Bran meal	2.9	0.9	0.01
Olive leaves	0	3	6
Olive leaves analytical composition (g kg ${}^{-1}$ )			
Metabolizable energy (kcal kg ${}^{-1}$ )	2713	2713	2713
Crude protein (%)	25.4	25.5	25.5
Ca (%)	1.26	1.26	1.26
P (%)	0.94	0.93	0.90
Lysine (%)	1.63	1.63	1.63
Methionine (%)	0.59	0.59	0.59
Premix growth 2 composition per kg			
Vitamin A	800 000 IU	–	–
Vitamin D ${}_{3}$	300 000 IU	–	–
Vitamin E	3000 mg	–	–
Vitamin B ${}_{1}$	100 mg	–	–
Vitamin K ${}_{3}$	200 mg	–	–
Vitamin B ${}_{2}$	500 mg	–	–
Vitamin B ${}_{6}$	300 mg	–	–
Vitamin B ${}_{12}$	15 mg	–	–
Niacin (ppm)	5000 mg	–	–
Pantothenic acid	1600 mg	–	–
Biotin	15 mg	–	–
Folic acid	100 mg	–	–
Iron	7000 mg	–	–
Copper	875 mg	–	–
Zinc	7000 mg	–	–
Manganese	12 250 mg	–	–
Iodine	175 mg	–	–
Cobalt	70 mg	–	–
Selenium	35 mg	–	–
Methionine	180 000 mg	–	–
Lysine	150 000 mg	–	–
Choline chloride	50 000 mg	–	–
Antioxidant	10 000 IU		

${}^{*}$ The composition of premix growth 2 per kg is shown in the lower section of this table.

### 2.4 Animals

All study procedures and guidelines for experimental animals were approved by the Animal Welfare and Experimentation Commission Blida (78 CBEEA-Blida: no. 28/SD/20190331). Broiler medium strain (BUT premium) female turkeys from the breeder Aviagen (*Meleagris gallopavo* turkeys, 270 animals) were used. All one-day-old poults were raised in a brooding house from birth to 3 weeks, with ad libitum access to feed and water. The animals were randomly placed in nine pens of approximately 15 m ${}^{2}$ of surface area, with a density of three turkeys per m ${}^{2}$ . To house the abovementioned groups, these nine pens were randomly divided into OL0, OL3, and OL6, each comprising 90 animals equitably distributed in three pens and three subgroup for each turkey-fed group. From the second week, the litter was made of chopped straw at a rate of 7 kg m ${}^{-2}$ , with a lighting program of 16 h d ${}^{-1}$ and a single 8 h dark period applied daily. According to the strain breed selector's recommendation, the light intensity was around 40 lux throughout the rearing period.

### 2.5 Determination of performance values

Beginning on the third week, the female turkeys were weighed individually every 14 d on a digital scale. Feed intake controls were performed at the same rate per pen by weighing the feed rejected before weighing the animals. The feed conversion ratio (FCR) was calculated by dividing the amount of feed consumed by the weight gain of the turkeys in each pen. Mortality inside the groups was recorded between the 3rd and 15th week of rearing.

### 2.6 Slaughter performance

Twenty-seven female turkeys (nine animals per group) were removed from their pens at the end of the experiment, placed into new ones, and allowed to fast for 12 h before being sacrificed. Live weights before slaughter, plucked turkey weight, carcass weight on the eviscerated animal, organ weight (liver, heart, proventriculus, gizzard, spleen), and abdominal fat were all measured. Carcass yield was calculated as carcass yield = (Eviscerated animal weight × 100) / Live weight.

### 2.7 Welfare observation indicators

At the end of the experiment, observations on the turkeys' welfare status were made on each animal, including feather cleanliness, tarsus lesions, and plantar lesions. Each criterion was scored by three observers on a scale of 0 to 4 (Supplement Fig. S1).

### 2.8 Determination of antioxidant activity

*Total antioxidant status*. At the end of the experiment, total antioxidant activity was performed on the serum samples of 18 animals (6 per group). Each test was repeated at least three times independently. The total antioxidant status was measured using the ABTS (2.2 ${}^{\prime }$ -azinobis-3-ethylbenzothiazoline-6-sulfonic acid di-ammonium salt) free radical discoloration test (Re et al., 1999). The ABTS solution has a blue coloration; it was solubilized in distilled water at a final concentration of 7 mM. The radical cation (ABTS + ) resulted from the reaction of the ABTS solution with a potassium persulfate at a final concentration of 2.45 mM. This mixture was incubated in the dark at room temperature for 12–16 h before use. In the present study, the ABTS + solution was diluted with phosphate buffer, pH 7.4, to an absorbance of 0.70 ± 0.02 at 734 nm. Total antioxidant status was performed on the serum samples of the control group, OL3 group, and OL6 group. After adding 1 mL of ABTS solution to 10 µ L of each sample (serum), the bluish coloration decreased inversely proportional to the antioxidant power of the antioxidant agent. The reading was taken exactly 6 min after the initial mixing. The inhibition percentage of ABTS at 734 nm was calculated. Each test was repeated at least three times independently.

*Effect on thiobarbituric acid reactive substances (TBARS) formation*. The assessment of the levels of TBARS was measured on serum samples of each animal at the end of the experiment. Each test was repeated at least three times independently. The thiobarbituric acid-reactive substances method assessed lipid peroxidation, which was based on the formation of a pink-colored complex resulting from the reaction between one malondialdehyde molecule and two thiobarbituric acid molecules. The complex formed absorbs at 532–535 nm (Devasagayam et al., 2003). The principle of this method is summarized in Supplement Fig. S2. Lipid peroxidation was assessed by determining TBARS formed in serum according to the previously described method (Devasagayam et al., 2003). The levels of TBARS were measured in the serum samples of the control group, OL3 group, and OL6 group. A sample suspension (1 mL of serum) was deproteinized by adding 0.5 mL TCA (trichloroacetic acid, 30 %) and incubated at 0 °C for 120 min, followed by centrifugation at 3000 rpm and 4 °C for 10 min. To 1 mL of the supernatant was added 0.25 mL of 1 % TBA (thiobarbituric acid, dissolved in 0.05 M of NaOH) and 0.075 mL of EDTA (ethylene diamine tetra acetic acid, 0.1 mol L ${}^{-1}$ ); then it was heated to 95 °C for 15 min followed by cooling. The absorbance of the colored product was measured at 535 nm. Each test was repeated at least three times independently.

### 2.9 Hematological and biochemical analyses

*Blood sampling*. Blood samples (approximately 5 mL) were collected from the wing veins of 27 animals (9 animals from each group) at 6, 10, and 14 weeks of age. Tubes containing EDTA were used for hematological analysis, and tubes containing lithium heparin were used for biochemical analysis. Blood samples were stored in a cooler centrifuged at 3000 rpm for 10 min, and the analyses were performed the same day.

*Hematological parameters*. Hematological analyses were performed with an automatic Mindray BC-30s. The parameters measured were white blood cells (denoted WBC), red blood cells (denoted RBC), hemoglobin (Hb), hematocrit (Hct), mean corpuscular hemoglobin concentration (MCHC), and platelets (Plt).

*Biochemical parameters*. Biochemical analyses were performed with a BioSystems BA200 (BioSystems S.A., Barcelona, Spain). The parameters measured were blood glucose (Gly), triglycerides (denoted TG), cholesterol (Chol), total serum protein (Prot T), and albumin (Alb).

**Figure 1:**
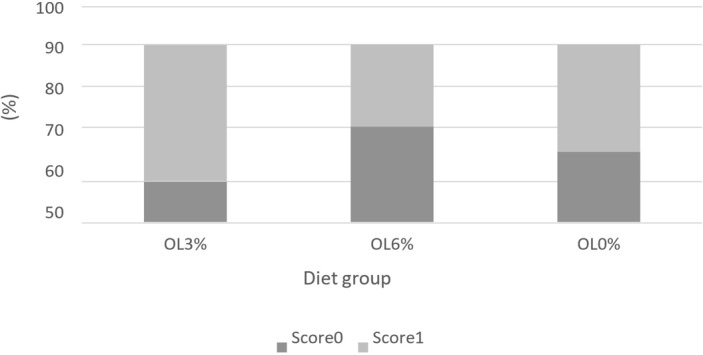
Scores of feather cleanliness according to diet (Welfare Quality, 2009).

**Figure 2:**
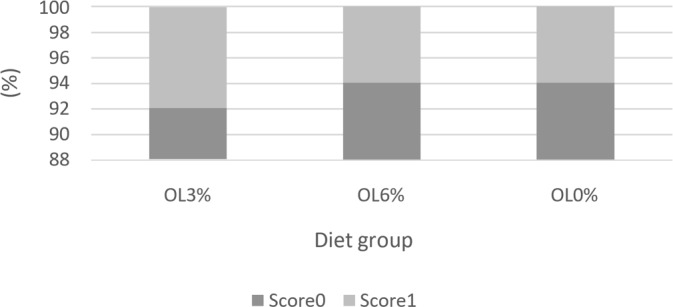
Scores of tarsal lesions according to diet (Welfare Quality, 2009).

**Figure 3:**
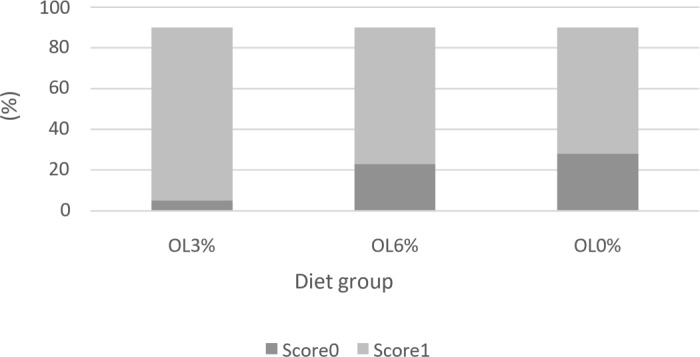
Scores of pododermatitis according to diet (Welfare Quality, 2009).

### 2.10 Statistical analyses

SAS (Statistical Analysis System) software was used for statistical analyses. Descriptive statistics such as arithmetic mean, maximum and minimum values, and standard deviation were obtained for the investigated parameters. The general linear model (GLM) procedure was used to analyze variance for growth performance weight and average daily weight gain. The following fixed linear model was used to estimate the effects of various factors on growth performance:

1 $$y_{ijk}=\mu +a_{i}+b_{j}+(ab{)}_{ij}+e_{ijkl},$$

where yijkl represent live weight and average daily weight gain of turkeys, as well as hematological and biochemical parameters. μ= mean, $a_{i}$ = effect of feed group, $b_{j}$ = effect of age of turkeys, $(ab{)}_{ij}$ = effect of age × group interaction, and $e_{ijkl}$ = random error.

Fisher's exact test was used to compare the mortality rates of the three groups studied. Analysis of variance was performed using the ANOVA procedure (PROC ANOVA, SAS) for hematological and biochemical parameters, as well as carcass yield and organ weight parameters.

## 3 Results

### 3.1 Zootechnical performance and welfare of turkeys

For the entire study period (3 to 15 weeks), the mortality rate in the three study groups was not significantly different ( p>0.05 ); for the OL0, OL3, and OL6 groups, it was 1.66 %, 4.16 %, and 1.66 %, respectively. The effects of group, sex, age, and sex × group × age interaction on turkey live weight (Table 5) and average daily weight gain (ADG) (Table 6) were highly significant ( p < 0.001). Animals in the OL3 group had significantly higher weights at the end of the experiment than the OL6 and OL0 groups (Table 5). Feed consumption for the whole experiment (3 to 13 weeks) was 11 727.52, 12 391.56, and 12 515.98 g respectively for groups OL0, OL3, and OL6.

**Table 5:** Effect of olive leaves incorporation on turkeys' growth (g).

Age (week)	Feed group (Ls means ± SE)			p value			$R^{2}$
	OL0 %	OL3 %	OL6 %	Age	Group	Age × group	
3	484.18 ± 7.32	477.34 ± 6.10	488.18 ± 6.35	< 0.001	< 0.001	< 0.001	0.97
7	2035.00 ± 35.85	2096.28 ± 24.70	2015.24 ± 20.42				
13	6563.67 ${}^{b}$ ± 102.79	6720.45 ${}^{a}$ ± 99.07	6599.85 ${}^{b}$ ± 112.31				

Mean values with different letters (a, b) are significantly different ( p < 0.05); $R^{2}$ : coefficient of determination. Ls represents least square means.

**Table 6:** Effect of olive leaves incorporation on average daily gain of turkeys (g).

Age (week)	Feed group (Ls means ± SE)			p value			$R^{2}$
	OL0 %	OL3 %	OL6 %	Age	Group	Age × group	
3–6	47.99 ± 2.40	50.81 ± 1.71	46.10 ± 1.49	< 0.001	< 0.001	< 0.001	0.54
7–13	62.78 ± 2.86	64.83 ± 2.75	62.98 ± 2.67				

Mean values with different letters (a, b) are significantly different ( p < 0.05); $R^{2}$ : coefficient of determination.

The OL0 group's feed conversion ratio was significantly lower ( p < 0.05) than the OL3 and OL6 groups (Table 7). Turkeys' final weight, carcass yield, organ weights, and carcass characteristics were compared by feed group at 15 weeks of age. The weights after bleeding and evisceration in the OL3 and OL0 groups were significantly higher ( p < 0.05) than in the OL6 group. The OL0 turkey group had significantly higher spleen, heart, and abdominal fat weights than the OL3 and OL6 groups. There were no significant differences ( p>0.05 ) in carcass yield, gizzard, or liver weights between the three experimental groups (Table 9).

Figures 1, 2, and 3 depict the welfare results of turkeys, demonstrating a lack of significant differences ( p>0.05 ) in feather cleanliness and tarsal lesion scores. Most turkeys (OL0: 75 %, OL3: 66.67 %, and OL6: 75 %) had clean feathers (Fig. 1). Furthermore, more than 90 % of the turkeys in all tested groups had tarsal lesions with a score of 0 (Fig. 2). In contrast, pododermatitis scores were predominantly classified as 1 in all groups. There was a trend of diet effect on pododermatitis scores ( p = 0.06). Indeed, the OL3 group (95.44 %) had a higher percentage of pododermatitis score 1 than the OL6 (72.22 %) and OL0 (69.44 %) groups (Fig. 3).

**Table 7:** Effect of olive leaves incorporation on feed conversion ratio.

Age (week)	Feed group (Ls means ± SE)			p value			$R^{2}$
	OL0 %	OL3 %	OL6 %	Age	Group	Age × group	
3–6	1.81 ± 0.02	1.91 ${}^{a}$ ± 0.02	1.95 ${}^{a}$ ± 0.02	< 0.001	< 0.001	< 0.001	0.59
7–13	2.59 ${}^{b}$ ± 0.03	2.68 ${}^{a}$ ± 0.03	2.73 ${}^{a}$ ± 0.03				

Mean values with different letters (a, b) are significantly different ( p < 0.05); $R^{2}$ : coefficient of determination.

**Table 8:** Effect of feed groups on body weight at slaughter, carcass yields, and internal organs weight.

Parameters	Feed group (Ls means)					
	OL0 %	OL3 %	OL6 %	SEM	p value	$R^{2}$
Final live weight (g)	8058.00 ${}^{a}$	8188.16 ${}^{a}$	7092.56 ${}^{b}$	181.70	0.0006	0.46
Weight after bleeding (g)	7806.92 ${}^{a}$	7868.00 ${}^{a}$	6830.11 ${}^{b}$	190.71	0.001	0.43
Eviscerated animal weight (g)	6279.17 ${}^{a}$	6405.17 ${}^{a}$	5511.00 ${}^{b}$	152.12	0.0008	0.45
Liver (g)	127.96	115.46	111.97	6.2	0.39	0.08
spleen (g)	10.49 ${}^{a}$	8.31 ${}^{b}$	8.51 ${}^{b}$	0.54	0.01	0.31
heart (g)	24.96 ${}^{a}$	23.74 ${}^{b}$	21.34 ${}^{b}$	1.17	0.02	0.25
Proventriculus (g)	11.53 ${}^{a}$	11.29 ${}^{\mathrm{ab}}$	10.28 ${}^{b}$	0.45	0.04	0.23
Gizzard (g)	106.97	114.76	114.25	16.78	0.38	0.08
Abdominal fat (g)	84.80 ${}^{a}$	74.61 ${}^{b}$	58.54 ${}^{c}$	8.76	0.09	0.17
Carcass yield (%)	77.90	78.22	77.68	0.44	0.63	0.08

Mean values with different letters (a, b, and c) are significantly different ( p < 0.05); SEM: standard error of the mean; $R^{2}$ : coefficient of determination.

### 3.2 Hematological parameters

Table 9 shows the effects of olive leaves on blood parameters at various ages. The OL3 group had a significantly lower ( p < 0.05) mean corpuscular hemoglobin concentration (MCHC) than the OL0 group (45.19 g dL ${}^{-1}$ ) or the OL6 group (45.75 g dL ${}^{-1}$ ). Other blood parameters showed no significant difference ( p>0.05 ) between the three experimental groups (Table 9). Table 10 shows the effects of olive leaves on serum biochemical parameters. The mean cholesterol level in the OL3 (1.30 g L ${}^{-1}$ ) and OL6 (1.29 g L ${}^{-1}$ ) groups was significantly ( p < 0.05) lower than in the OL0 (1.39 g L ${}^{-1}$ ) group (Table 11). The albumin levels in the OL3 (13.32 g L ${}^{-1}$ ) and OL6 (12.90 g L ${}^{-1}$ ) groups were comparable ( p>0.05 ). It was, however, significantly different ( p < 0.05) between the OL6 and OL0 groups (13.67 g L ${}^{-1}$ ). For the other biochemical parameters (Table 11), no significant difference ( p>0.05 ) was found between the three experimental groups.

**Table 9:** Blood parameters of turkeys for different groups according to the diet.

Parameters	Age (weeks)	Groups			Statistical effects				
		OL0 %	OL3 %	OL6 %	SEM	Group (Gr)	Age	Gr × Age	$R^{2}$
WBC (10 ${}^{3}$ )	6	112.19	115.77	110.37	2.36				
	10	118.80	114.57	113.36		0.16	0.35	0.07	0.17
	14	118.11 ${}^{b}$	109.35 ${}^{a}$	115.60 ${}^{b}$					
	Total	116.37	113.23	113.11	1.37				
RBC (10 ${}^{6}$ )	6	2.20	2.29	2.21	0.06				
	10	2.28	2.20	2.14		0.31	0.54	0.58	0.08
	14	2.30	2.26	2.22					
	Total	2.26	2.25	2.19	0.03				
HB (g dL ${}^{-1}$ )	6	14.01	14.62	14.14	0.34				
	10	15.08	14.50	14.36		0.32	> 0.001	0.38	0.38
	14	16.29	15.72	15.61					
	Total	15.13	14.95	14.70	0.20				
Hct (%)	6	31.57	32.70	31.46	0.90				
	10	32.04	31.58	30.60		0.13	> 0.001	0.63	0.41
	14	37.11 ${}^{b}$	35.68 ${}^{\mathrm{ab}}$	34.90 ${}^{a}$					
	Total	33.57	33.38	32.18	0.52				
MCV (fl)	6	143.59	142.74	142.43	1.91				
	10	140.86	143.60	142.71		0.44	> 0.001	0.29	0.67
	14	161.60	158.89	155.30					
	Total	148.68	148.41	146.81	1.10				
MCHC (pg)	6	44.53	44.80	45.02	0.42				
	10	47.14	45.97	46.94		0.03	> 0.001	0.21	0.48
	14	43.91	43.84	45.28					
	Total	45.19 ${}^{\mathrm{ab}}$	44.87 ${}^{a}$	45.75 ${}^{b}$	0.24				

WBC: white blood cells. RBC: red blood cells. Hb: hemoglobin. Hct: hematocrit. MCHC: mean corpuscular hemoglobin concentration. MCV: mean corpuscular volume. Mean values with different letters (a, b, and c) are significantly different ( p<0.05 ); SEM: standard error of the mean; $R^{2}$ : coefficient of determination.

**Table 10:** Effect of turkey's diet on biochemical parameters.

Parameters	Age (weeks)	Groups			Statistical effects				
		OL0 %	OL3 %	OL6 %	SEM	Group (Gr)	Age	Gr × Age	$R^{2}$
Gly (g L ${}^{-1}$ )	6	3.75	3.77	3.50	0.08				
	10	3.41	3.48	3.60		0.97	< 0.001	0.02	0.52
	14	3.17	3.12	3.27					
	Total	3.44	3.46	3.46	0.04				
TG (g L ${}^{-1}$ )	6	0.48	0.41	0.66	0.18				
	10	0.75	0.63	0.50		0.97	< 0.001	0.51	0.35
	14	1.16	1.44	1.31					
	Total	0.79	0.83	0.82	0.10				
Chol (g L ${}^{-1}$ )	6	1.32	1.39	1.30	0.06				
	10	1.44	1.28	1.16		0.09	0.46	0.02	0.21
	14	1.42 ${}^{b}$	1.23 ${}^{a}$	1.42 ${}^{b}$					
	Total	1.39 ${}^{b}$	1.30 ${}^{a}$	1.29 ${}^{a}$	0.03				
Prot T (g L ${}^{-1}$ )	6	31.27	29.18	30.30	1.82				
	10	45.05	43.29	47.16		0.12	< 0.001	0.87	0.64
	14	31.79	29.53	33.79					
	Total	36.03 ${}^{\mathrm{ab}}$	34.00 ${}^{a}$	37.08 ${}^{b}$	1.05				
Alb (g L ${}^{-1}$ )	6	10.57	10.43	9.67	0.41				
	10	13.32	13.41	12.49		0.13	< 0.001	0.31	0.84
	14	17.12	16.11	16.79					
	Total	13.67 ${}^{a}$	13.32 ${}^{\mathrm{ab}}$	12.98 ${}^{b}$	0.24				

Mean values with different letters (a and b) on the same line indicate significant differences ( p<0.05 ); SEM: standard error of the mean; $R^{2}$ : coefficient of determination; Gr: Group; A: Age in weeks. Gly: glycemia; TG; triglyceride; Chol: cholesterol; Prot: protein; Alb: albumen

### 3.3 Oxidative status

This study assessed the oxidative status of the three turkey groups by measuring total antioxidant status (TAS) and determining the levels of thiobarbituric acid reactive substances (TBARS). The values in Table 11 indicate that treatment with OLs at 3 % and 6 % resulted in significantly higher TAS in turkey serum compared to the control group. TBARS values were also significantly lower in the OL3 and OL6 groups than in the OL0 group (Table 9). Adding olive leaves (OLs) reduced TBARS content by 3 % and 6 %, respectively.

**Table 11:** Oxidative status per group.

Parameters	Groups			Statistical effects		
	OL0 %	OL3 %	OL6 %	SEM	Group	$R^{2}$
TAS (%)	82.6	87.3	87.5	0.70	0.0002	0.67
TBARS (nm)	0.392	0.176	0.170	0.22	< 0.001	0.80

Mean values with different letters (a and b) are significantly different ( p<0.05 ); SEM: standard error of the mean; $R^{2}$ : coefficient of determination

## 4 Discussion

### 4.1 Zootechnical performance and welfare of turkeys

The different diets supplemented with olive leaves (OL) had no effect on mortality. Several studies on broilers-fed diets supplemented with 0.7 % and 1 % OL (Sarica and Ürkmez, 2016) or OL extracts (Erener et al., 2020) found similar results. On the other hand, authors using OL powder (Varmaghany et al., 2013) or OL extracts (Jabri et al., 2017) reported that mortality in broilers fed OL-supplemented feed was significantly reduced. The effect of dietary supplementation with OL at different concentrations revealed that the OL3 group showed greater weight growth than the OL6 and OL0 groups ( p < 0.001). However, Govaris et al. (2010) found that mean body weights and feed conversion rates did not differ significantly between groups of turkeys fed diets supplemented with 10 g kg ${}^{-1}$ olive leaves. Botsoglou et al. (2010) reported similar results when supplementing turkeys with 5 and 10 g of OL powder per kilogram of feed, indicating that these incorporation rates have no effect on turkey growth.

There is a scarcity of research studies that have employed OL powder or OL extracts in the context of turkey feed. Several studies, however, have been conducted on *Gallus gallus* in this regard. In both broiler chicken (Ait-Kaki et al., 2018; El-Damrawy et al., 2013; Erener et al., 2020) and laying hens (Cayan and Erener, 2015), the authors reported positive effects on growth, feed conversion ratio, and carcass yield. Notably, dietary supplementation of laying hens with OL 3 % powder did not affect feed conversion or laying rates but increased the hen's final body weight. In parallel, feed consumption was lowest in the OL0 group. This result may be justified by the live weight of the animals in this group and their low feed conversion ratio. The average daily weight gain of turkeys in the current study was significantly higher in OL3 and OL6 compared to OL0 (Table 6). This could be attributed to an improvement in flavor, changes in caecal microflora, and the beneficial effect of moderate amounts of different fiber sources in the diet, which aids in developing the chicken's digestive organs (Gonzalez-Alvarado et al., 2007).

Plant extracts also affect digestion and the secretion of digestive enzymes (Platel and Srinivasan, 2000). Furthermore, polyphenolic compounds in OL help eliminate pathogenic microorganisms that could potentially develop in digestive organs, prevent toxin effects from feed, and stimulate digestive enzymes. OL is a viable alternative to growth promoter feed additives (Erener et al., 2020). Similarly, Erener et al. (2020) found that the treatments had no effect on *Lactobacillus* spp., which are beneficial microorganisms. However, compared to the control group, all levels of OLs had a significant antibacterial effect on *Clostridium* spp. and *Staphylococcus aureus* populations.

Oleuropein levels of 50, 100, and 150 mg kg ${}^{-1}$ improved laying hen production, egg mass, and feed conversion rate (Erener et al., 2020). Furthermore, it has been reported that the antioxidant properties of active compounds in OL can improve nitrogen retention and, as a result, bird growth by reducing protein oxidation (El-Hakim et al., 2009). The weight of animals in the OL6 group at slaughter (15 weeks) was significantly lower than that of the OL3 and OL0 groups, according to the findings. Adding 5 % or 10 % OL to growing pig diets has also resulted in altered growth rates and feed conversion ratios (Paiva-Martins et al., 2009). In the OL3 and OL6 groups, compared to the OL0 group, OL incorporation resulted in a significant decrease in abdominal fat and an increase in FCR, with an inversely proportional effect to the amount of OL incorporated. According to Shafey et al. (2013), OL supplementation had no effect on abdominal fat levels.

Olive pomace is high in fiber (lignin, cellulose, hemicellulose, and pectin) and unsaturated fatty acids; it contains phenolic, polyphenols, oleuropeosides, and flavonoids (Yeniçeri et al., 2021). In birds fed diets containing up to 7.5 % olive pomace (with 10.2 % crude protein (CP) and 12 % ether extract (EE)), increasing levels of olive pomace (0 % to 10 %) resulted in weekly lower weight gain. The high crude fiber content (6.1 %) was attributed to the result (Raayaa et al., 2011). In its structure, olive pulp (OP) contains a variety of active compounds (antioxidant, antifungal, and antibacterial) (Ahmed et al., 2014; Alves et al., 2019). According to Sayehban et al. (2020), adding 5 % and 10 % olive oil pulp to a broiler diet does not change the carcass characteristics. Furthermore, Sateri et al. (2017) found no significant difference in broiler chicken growth performance when 0 %, 2 %, 4 %, 6 %, and 8 % OP were added to feed. In this last study, olive pomace yielded high growth performance without requiring enzyme supplementation.

Animal welfare encompasses various aspects ranging from health and physical well-being as well as psychological well-being to the ability to express physiological and species-specific behaviors. Several indicators are inextricably linked to poultry welfare and farm economic returns (FAWC, 2011). The parameters chosen for this study are physical welfare indicators with lesion scores affecting various anatomical parts. In this context, Welfare quality (2009) proposed using a scale with different scores for the classification of several animal welfare indicators, such as feather cleanliness, pododermatitis, and lameness, in the European project Welfare Quality^®^.

Tarsal lesion scores in the current study were identical across the three experimental groups, with a score of 0 indicating complete absence of tarsal lesions. Similarly, no statistical difference in plantar lesion scores was found between groups. Most turkeys received a score of 1, indicating they had low plantar lesions. Under the rearing conditions, the turkeys must have lesion scores of 0 and/or 1 to avoid the locomotion problems frequently reported in fast-growing broiler turkey farms, which reduce animal movement and affect animal welfare and, thus, production performance. In this regard, a study in a German commercial farm with a capacity of 11 860 turkeys found that 60 % of the females and 33.8 % of the males had plantar lesions at 16 weeks of age (Krautwald-Junghanns et al., 2011). These pododermatitis lesions, which include inflammation and necrotic damage on the plantar surface, are common in broiler and fast-growing turkey flocks (Shepherd et Fairchild, 2010).

Various factors can cause pododermatitis and tarsal lesions, the most common of which is feeding. Diarrhea caused by unbalanced feed rations can moisten and degrade the litter, promoting contact between the tarsus and the feet with solid soil and thus initiating the primary stages of the lesions. In this regard, Kaukonen et al. (2016) reported that maintaining dry bedding is critical for poultry footpad health. Regarding feather cleanliness, the three groups of turkeys scored similarly, with clean feathers indicating clean litter. When droppings are wet and/or sticky, they easily stick to the animal's body, promoting the development of body lesions (Eichner et al., 2007). These lesions directly impact the turkeys' well-being by causing pain and reducing the movement required to water and feed. This inhibits the expression of natural behaviors and the production potential (Jensen and Toates, 1993).

### 4.2 Blood parameters

Hematological parameters play an important role in detecting health changes that may be missed by physical examination, such as ascites pathology in poultry, particularly broiler chickens (Scheele et al., 2003). Almost all blood parameters in the current study showed no significant difference ( p>0.05 ) between the three experimental groups, except for mean corpuscular hemoglobin concentration (MCHC), which was significantly lower in the OL3 group compared to the OL0 and OL6 groups (44.87 vs. 45.19 and 45.75 g dL ${}^{-1}$ , respectively) (Table 9). In the study of Varmaghany et al. (2013), the effect of dietary olive leaves supplementation on broiler hematological parameters (including systolic blood pressure, packed cell volume, alanine aminotransferase, erythrocyte osmotic fragility, red blood cell count, and triiodothyronine level) was more obvious under cold stress compared to standard conditions, thereby ameliorating ascites disease syndrome.

Some blood metabolites, on the other hand, such as high-density lipoprotein (HDL), low-density lipoprotein (LDL), total cholesterol, and triglycerides (metabolites of hepatic lipid metabolism), reflect the metabolic status of the animal and inform if the food consumed acts in a toxic way, which can cause health problems and harm animal productivity (de Oliveira et al., 2021). In the current study, plasma cholesterol levels in turkeys from the OL3 and OL6 groups were significantly lower ( p < 0.05) than in the OL0 group. Similar findings have been reported in the Japanese quail (Christaki, 2011; Sarica and Toptas, 2014). Furthermore, blood cholesterol levels in broilers supplemented with OL were found to be lower (Parsaei et al., 2014). On the other hand, Zangeneh and Torki (2011) found no effect on laying hens.

Coni et al. (2000) and Andreadou et al. (2006) attributed the reduction in cholesterol in rabbits fed a diet enriched with OLs to the cholesterol-lowering effect of oleuropein and the hydroxytyrosol-rich extracts of this plant. On the other hand, Krzeminski et al. (2003) suggest that the cholesterol-lowering property of OLs may be related to reduced intestinal absorption of cholesterol or decreased liver synthesis. Furthermore, Prasad and Kalra (1993) discovered that OLs stimulate biliary cholesterol secretion and excretion in the feces. Poernama et al. (1992) found that maintaining normal levels or lowering levels of this metabolite, along with LDL and triglycerides, in the blood plasma of birds reduces the likelihood of atherosclerosis disease developing. Lowering cholesterol in chickens, on the other hand, is not a goal because cholesterol is required for proper fat digestion and cell membrane stability (Erener et al., 2019).

Here, the different OL supplements had no effect on the serum concentrations of glucose and triglycerides. However, several authors have noted that the OL hypoglycemic effects decrease plasma triglycerides in blood plasma (Christaki, 2011; Jemai et al., 2008; Nafea and Hussein, 2018). Furthermore, Ait-Kaki et al. (2018) reported a significant decrease in broiler chicken cholesterol that was not accompanied by effects on triglycerides or total plasma proteins.

### 4.3 Oxidative status

Previous studies have reported OL extracts' ABTS scavenging activity (Bouaziz and Sayadi, 2005; Orak et al., 2012; Kiritsakis et al., 2010). OLs are rich in polyphenols and flavonoids, which have antioxidant properties and involve various biological processes and mechanisms, including preventing or reducing oxidative damage (Kočevar et al., 2013; Vickers et al., 2017). The current findings are consistent with those reported by Govaris et al. (2010), who found that olive leaves incorporated in turkey feed at 10 g kg ${}^{-1}$ are more effective in inhibiting lipid oxidation than rosemary at the same dose but less effective than 300 mg kg ${}^{-1}$ of α -tocopherol. Botsoglou et al. (2010) found that including OLs in turkey diets at 5 and 10 g kg ${}^{-1}$ significantly delayed lipid oxidation in chilled breast fillets.

El-Damrawy et al. (2013) reported the same results in chickens supplemented with OL powder and rabbits using olive leaf extract. Compared to the control group, the authors found very low levels of TBARS. The results of the present study are also similar to those of Paiva-Martins et al. (2009). In addition, the authors reported that the meat of turkeys and pigs receiving olive leaves had better oxidative stability. The inhibition of lipid oxidation after dietary supplementation with olive leaves is certainly the result of various constituents, mainly phenolics and flavonoids, which have antioxidant properties capable of scavenging free radicals (Benavente-Garcia et al., 2000). In previous studies, the addition of OL to turkey diets led to increased serum concentrations of polyphenols such as oleuropein, hydroxytyrosol, carotene, triglycerides, tocopherol, and sitosterol that are mainly responsible for antioxidant activity and protection from blood lipid oxidation (Lins et al., 2018; Moudache et al., 2016).

## 5 Conclusions

The findings reported in this paper indicate that incorporating 3 % and 6 % olive leaves in turkeys' diet results in similar and statistically significant effects on various aspects, including growth, blood and serum biochemical parameters, welfare, and oxidative status. Nevertheless, it seems that incorporating a 3 % olive leaf supplementation into turkeys' diet holds greater potential for enhancing the weights of live animals and their carcasses following the processes of bleeding and evisceration. Further investigation is warranted to ascertain the impact of olive leaves on poultry nutrition under varying environmental conditions, particularly in the context of cold stress. Additionally, it remains to be determined whether the inclusion of enzymes for fiber digestion and supplementation of amino acids are necessary to attain an optimal outcome for bird growth. Ultimately, OL can be a feasible substitute for feed additives employed in poultry diets to enhance growth. In order to sustain optimal growth and health outcomes, it is imperative to employ suitable processing techniques that safeguard the aroma, digestibility, and nutrient composition of animal feed.

## Supplement

10.5194/aab-67-163-2024-supplement The supplement related to this article is available online at: https://doi.org/10.5194/aab-67-163-2024-supplement.

## Data Availability

The data can be found in the article (tables, figures, sections, etc.).

## Tribute

Sadoudi Ahmed, a graduate veterinarian from the National Veterinary School (NVS) of Algiers in 2004, embarked on a doctoral thesis at the Faculty of Veterinary Medicine of the University of Liège starting from the academic year 2018/2019. He successfully completed his doctoral training, defending his thesis in 2021 with a grade of 17/20 (Highest Distinction). His thesis topic focused on the effects of incorporating olive leaf and/or nettle in feed on the zootechnical performance and welfare of broiler turkeys, a subject of great interest from economic, scientific, and ethical perspectives.

Ahmed was an exceptional researcher, eager for continuous education to strive for excellence. His joviality and kindness enabled him to forge friendships with doctoral students and researchers from various disciplines and nationalities. In 2022, Ahmed began a long battle against illness. He passed away on 29 March 2024, at the age of 45.

Ahmed leaves behind a wife and two children, Yasmine and Abdeslam. His death deeply affected all those who knew him at the University of Liège, his thesis supervisors, Belgian and international doctoral students enrolled in the Faculty of Veterinary Medicine, and the entire Department of Veterinary Management of Animal Resources of the Faculty of Veterinary Medicine. He leaves a scientific legacy with an original research article and several presentations at conferences and scientific seminars. His work will be invaluable for those who follow in his footsteps. Thank you, Ahmed.
