# Supplementary material for: Effect of olive leaf incorporation in animal feed on broiler turkey (Meleagris gallopavo) growth performance, welfare, oxidative status, and blood and biochemical serum parameters
Source: Arch Anim Breed. 2024 Apr 17;67(2):163–76. doi: 10.5194/aab-67-163-2024 (PMC13138611; doi:10.5194/aab-67-163-2024)
Supplement: The supplement related to this article is available online at: https://doi.org/10.5194/aab-67-163-2024-supplement. [file aab-67-163-2024-supplement.pdf]

Supplement of Arch. Anim. Breed., 67, 163–176, 2024
https://doi.org/10.5194/aab-67-163-2024-supplement
© Author(s) 2024. CC BY 4.0 License.

Open Access
Archives Animal Breeding

Supplement of

Effect of olive leaf incorporation in animal feed on broiler turkey (*Meleagris gallopavo*) growth performance, welfare, oxidative status, and blood and biochemical serum parameters

Ahmed Sadoudi et al.

Correspondence to: Nassim Moula (nassim.moula@uliege.be)

The copyright of individual parts of the supplement might differ from the article licence.

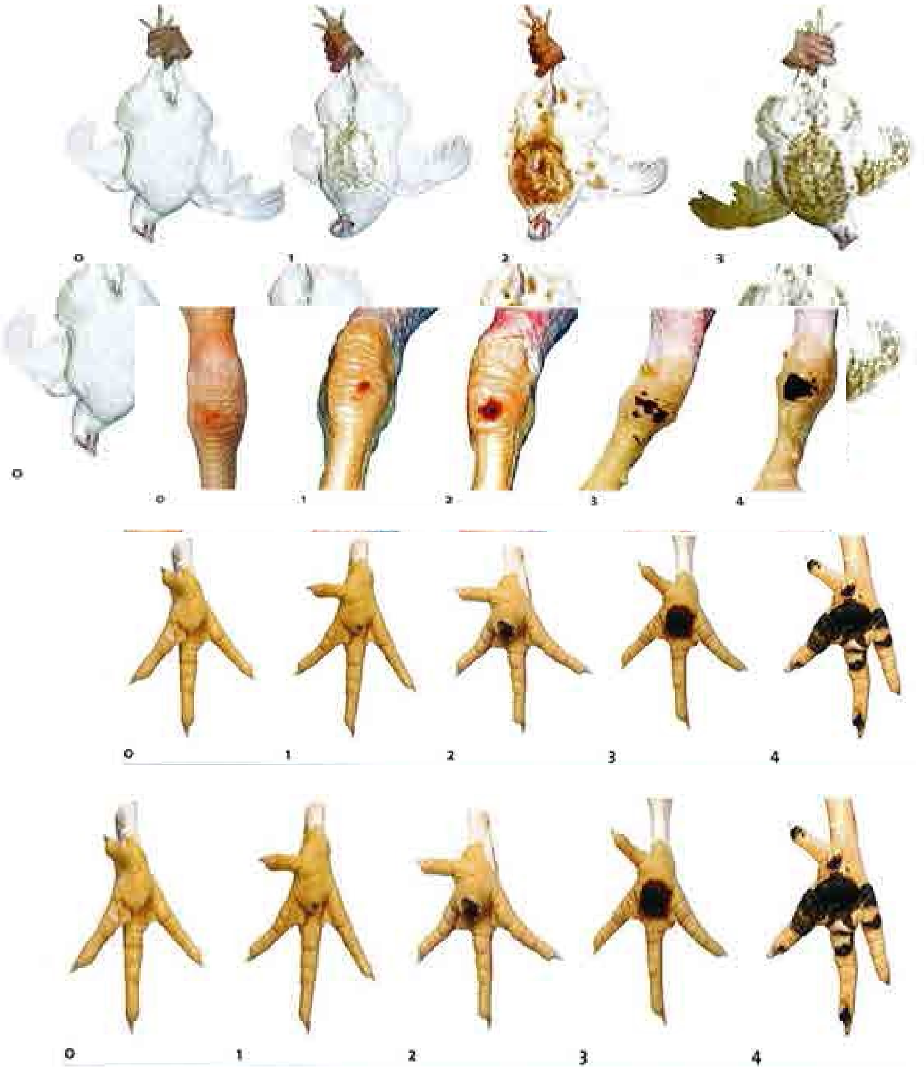

Figure S1. Scoring scale for leg lesions and feather cleanliness (Welfare Quality, 2009)

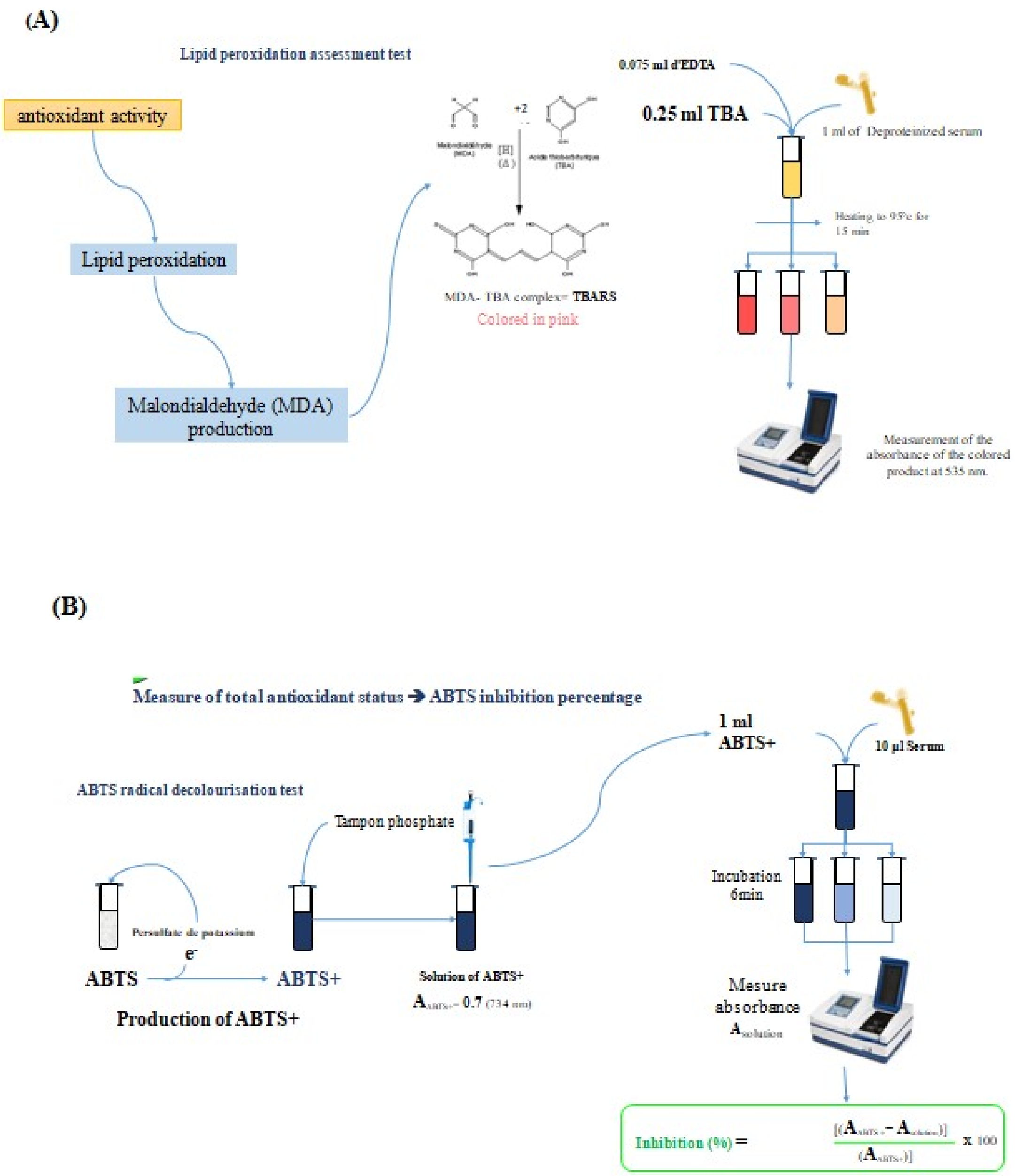

Figure S2. Lipid peroxidation assessment test (A); Measure of total antioxidant status (B)
